# From microbes to medicine: harnessing the power of the microbiome in esophageal cancer

**DOI:** 10.3389/fimmu.2024.1450927

**Published:** 2024-11-12

**Authors:** Xiaoyan Liu, Bang Li, Liping Liang, Jimin Han, Shijie Mai, Le Liu

**Affiliations:** ^1^ Department of Gastroenterology, The Second Affiliated Hospital of Guangzhou Medical University, Guangzhou, China; ^2^ Department of Gastroenterology, Zhujiang Hospital, Southern Medical University, Guangzhou, China; ^3^ Department of Gastroenterology and Hepatology, Guangzhou Key Laboratory of Digestive Diseases, Guangzhou Digestive Disease Center, Guangzhou First People’s Hospital, School of Medicine, South China University of Technology, Guangzhou, China; ^4^ School of Life Sciences, Tsinghua University, Beijing, China; ^5^ Department of Thoracic Surgery, Nanfang Hospital, Southern Medical University, Guangzhou, China; ^6^ Integrated Clinical Microecology Center, Shenzhen Hospital, Southern Medical University, Shenzhen, China

**Keywords:** esophageal cancer, microbiota dysbiosis, porphyromonas gingivalis, fusobacterium nucleatum, drug resistance, precision oncology

## Abstract

Esophageal cancer (EC) is a malignancy with a high incidence and poor prognosis, significantly influenced by dysbiosis in the esophageal, oral, and gut microbiota. This review provides an overview of the roles of microbiota dysbiosis in EC pathogenesis, emphasizing their impact on tumor progression, drug efficacy, biomarker discovery, and therapeutic interventions. Lifestyle factors like smoking, alcohol consumption, and betel nut use are major contributors to dysbiosis and EC development. Recent studies utilizing advanced sequencing have revealed complex interactions between microbiota dysbiosis and EC, with oral pathogens such as Porphyromonas gingivalis and Fusobacterium nucleatum promoting inflammation and suppressing immune responses, thereby driving carcinogenesis. Altered esophageal microbiota, characterized by reduced beneficial bacteria and increased pathogenic species, further exacerbate local inflammation and tumor growth. Gut microbiota dysbiosis also affects systemic immunity, influencing chemotherapy and immunotherapy efficacy, with certain bacteria enhancing or inhibiting treatment responses. Microbiota composition shows potential as a non-invasive biomarker for early detection, prognosis, and personalized therapy. Novel therapeutic strategies targeting the microbiota—such as probiotics, dietary modifications, and fecal microbiota transplantation—offer promising avenues to restore balance and improve treatment efficacy, potentially enhancing patient outcomes. Integrating microbiome-focused strategies into current therapeutic frameworks could improve EC management, reduce adverse effects, and enhance patient survival. These findings highlight the need for further research into microbiota-tumor interactions and microbial interventions to transform EC treatment and prevention, particularly in cases of late-stage diagnosis and poor treatment response.

## 1 Introduction

Esophageal cancer (EC) can be divided into two major histological subtypes: esophageal adenocarcinoma (EAC) and esophageal squamous cell carcinoma (ESCC) (1). ESCC predominantly arises in the upper to middle esophageal regions, whereas EAC is generally localized to the lower esophagus, particularly near the gastroesophageal junction (2). The prevalence of ESCC is significantly higher in regions such as Asia and Africa, while the incidence of EAC has been increasingly reported in Western countries over recent years (3). EC is currently the eighth most common and the sixth most deadly cancer globally (4). The progression of EC symptoms varies with the stage of the disease; early stages may be asymptomatic or involve dysphagia, progressing to more severe symptoms such as persistent pain and significant weight loss in advanced stages. Early-stage EC is often managed with endoscopic therapy, whereas locally advanced cases typically require a combination of chemotherapy and radiation, either before or after surgical intervention (5). For metastatic disease, a regimen of multiple therapies is employed to relieve symptoms and decelerate disease progression. Neoadjuvant treatments have been effective in improving survival rates, especially for ESCC, establishing them as a standard care approach for locally advanced cases (6). Risk factors for EC include chronic alcohol consumption, tobacco use (particularly smoking), gastroesophageal reflux disease (GERD), obesity, diets lacking in fruits and vegetables, previous radiation therapy to the chest or upper abdomen, Barrett’s esophagus (BE), achalasia, a history of certain cancers (e.g., head and neck or lung cancer), exposure to chemicals or asbestos, genetic predispositions, advanced age, and poor oral health (7, 8).

The human microbiota, comprising over 100 trillion microorganisms primarily residing on epithelial surfaces such as the skin, digestive, and respiratory tracts, plays dual roles in gastrointestinal tumor promotion and antitumor immune suppression (9). The gut microbiome, as the most diverse microbial community, is crucial for immune modulation and overall health by metabolizing beneficial compounds through dietary fiber digestion, vitamin synthesis, and short-chain fatty acid**s** (SCFA**s**) production (10). Advances in microbial analysis, such as 16S amplicon sequencing, shotgun sequencing, and multi-omics technologies, have enhanced our understanding of microbial communities within the host. These studies highlight the microbiome’s essential role in immune regulation, metabolism, growth, and cognition. The microbiome can also contribute to tumor progression via mechanisms like DNA damage, inflammation, immune disruption, modulation of cancer pathways, and carcinogen production. It significantly influences cancer treatment efficacy by modulating the host’s response to chemotherapy and immunotherapy. Despite homogeneity in key driver mutations in EC, phenotypic tumor heterogeneity is largely shaped by external factors, including the microbiota. Gut dysbiosis is a notable feature in EC patients, affecting inflammation and modulating treatment responses directly or via microbiota-derived metabolites. Advanced sequencing technologies and methods like fluorescence *in situ* hybridization have greatly improved our understanding of the microbiota’s role in EC, particularly in preclinical models. However, the specific contributions of microorganisms from different regions of the digestive tract to EC development remain unclear. Moreover, the invasive nature of esophageal sample collection poses challenges to studying the esophageal microbiome (11). This review provides a comprehensive overview of current knowledge on the microflora from various regions in both healthy individuals and EC patients, analyzing their impact on EC treatment. It also addresses challenges in the field and explores potential microbiota-driven strategies to improve therapeutic outcomes for EC patients.

## 2 Influence of microbiota across oral, esophageal, and intestinal ecosystems on esophageal carcinogenesis

### 2.1 Composition of the oral microbiota in EC

Recent studies have suggested a potential association between oral microbiota imbalance and digestive cancer development, including EC (12). Specific oral bacteria, such as Porphyromonas gingivalis and Streptococcus anginosus, are frequently detected in EC tissue. Moreover, various studies have identified increased levels of pathogenic bacteria in the saliva of EC patients (13, 14). Kawasaki et al. explored the link between periodontal pathogens and EC by analyzing subgingival plaque and saliva samples from both EC patients and healthy controls, finding that Tannerella forsythia and Streptococcus anginosus in dental plaque, as well as Aggregatibacter actinomycetemcomitans in saliva, were strongly associated with EC, suggesting a role for these pathogens in its pathogenesis (15). Similarly, Peters et al. observed that Tannerella forsythia was linked to an increased risk of EAC, while Porphyromonas gingivalis was associated with a higher risk of ESCC; conversely, reduced levels of Neisseria and Streptococcus pneumoniae were correlated with a lower risk of EAC (16). A study in China examined oral microbial diversity in ESCC patients using 16S rRNA sequencing and found significant differences compared to healthy individuals, particularly in microbial composition at the phylum and genus levels. ESCC patients had higher proportions of Firmicutes and Bacteroidetes, along with distinct differences in genera such as Streptococcus and Prevotella_7 (17). These findings underscore the necessity for further longitudinal and mechanistic studies to comprehensively elucidate the connection between oral microbiota and ESCC. Periodontal pathogens have emerged as key contributors to carcinogenesis through diverse mechanisms that are increasingly being elucidated. Notably, certain bacteria that are well known for causing periodontal diseases may also contribute to tumor formation in the esophagus. For instance, Fusobacterium nucleatum has been shown to promote the growth, migration, and invasion of cancer cells, thereby facilitating the initiation and progression of gastrointestinal malignancies (18). In EC tissues, notably higher concentrations of Fusobacterium nucleatum DNA were found, which correlated with shorter survival rates compared to normal esophageal mucosa (19). Porphyromonas gingivalis has been linked to EC development, as it invades oral epithelial cells, disrupts apoptosis, and promotes uncontrolled cell growth. By activating Toll-like receptors (TLRs) and the NF-κB pathway, this bacterium induces chronic inflammation through the release of inflammatory mediators. Such persistent inflammation contributes to DNA damage and genetic mutations, ultimately resulting in malignant transformation of esophageal epithelial cells. Research has demonstrated a significant correlation between Porphyromonas gingivalis infection and ESCC, indicating its potential role in tumor progression (20). The dysbiosis of the oral microbiota in EC patients is characterized by an increased abundance of pathogenic bacteria like Fusobacterium nucleatum and Porphyromonas gingivalis, alongside a decrease in beneficial microbial species. This imbalance creates a pro-inflammatory environment that facilitates carcinogenesis. The inflammatory mediators released in response to these pathogenic bacteria promote oxidative stress and DNA damage in host cells. Additionally, these bacteria can produce virulence factors, such as proteases and endotoxins, that degrade the extracellular matrix and enhance the invasive potential of cancer cells. Moreover, the oral cavity serves as a reservoir for these pathogens, which can translocate to the esophagus through swallowing or aspiration. Once in the esophageal mucosa, they can directly interact with epithelial cells and the local immune system, exacerbating inflammation and contributing to neoplastic changes. The ability of these bacteria to modulate immune responses allows them to create a microenvironment that is conducive to tumor growth and immune evasion. Understanding the specific alterations in the oral microbiota of EC patients provides valuable insights into the mechanisms underlying esophageal carcinogenesis. It highlights the importance of oral health and the potential role of periodontal pathogens in cancer development. Targeting these pathogenic bacteria through improved oral hygiene practices, antimicrobial therapies, or the use of probiotics may offer novel strategies for preventing EC or inhibiting its progression. In summary, oral microbiota in EC patients shows significant changes, particularly an increase in periodontal pathogens like Fusobacterium nucleatum and Porphyromonas gingivalis. These bacteria facilitate the development and progression of EC through mechanisms involving inflammation, immune modulation, and direct effects on epithelial cell growth and survival. Further research into these microbial interactions and mechanisms is essential for developing new diagnostic biomarkers and therapeutic interventions aimed at modulating the oral microbiota to prevent or treat EC.

### 2.2 Composition of the esophageal microbiome in EC

The esophagus is a muscular tube that connects the pharynx to the stomach, enabling the transit of food and liquids (21). In the 1980s, pioneering studies overturned the belief that the esophagus was sterile by demonstrating the existence of resident microbiota through culture-based methods (22). Compared to other parts of the gastrointestinal tract, the esophagus has a relatively sparse microbial community. Recent advancements in metagenomics have enhanced our understanding of the esophageal microbiota in healthy individuals, though sample collection still poses greater challenges compared to other anatomical regions. In 2004, Blaser et al. discovered 95 bacterial species across six phyla in the distal esophagus of healthy individuals using 16S amplicon sequencing, encompassing Firmicutes, Bacteroidetes, Fusobacteria, Proteobacteria, Actinobacteria, and TM7 (23). The predominant bacteria found in healthy esophageal microbiota is Streptococcus. In 2009, Zhiheng Pei et al. identified two distinct types of esophageal microbiota: Type I, dominated by Streptococcus in a phenotypically normal esophagus, and Type II, which includes a higher proportion of G**-**anaerobic/microaerophilic bacteria, often associated with esophagitis and BE (24). The unique anatomical location of the esophagus makes its microbiota particularly susceptible to external influences. Norder Grusell et al. confirmed that the microbiota of the upper, middle, and lower esophagus are similar, yet vulnerable to influences from adjacent structures (25, 26). The administration of medications such as proton pump inhibitors (PPIs) and antibiotics can significantly alter the esophageal microbiota, with notable changes observed following PPIs therapy (27), and antibiotic treatment in mouse models (28). Different sampling techniques used in the esophagus profoundly impact the study of its microbiota. Cell sponge sampling, a novel approach, yields microbial DNA quantities more than ten times greater than those obtained via esophageal brushing or biopsy, as measured by quantitative PCR. This method reveals a Lactobacillus enrichment within the EC microenvironment, albeit with reduced microbial diversity compared to control groups (11). The microbial communities associated with various esophageal diseases differ markedly from those in healthy esophagi (29). Abnormal esophageal microbiota, rich in G**-**rods such as Veillonella, Prevotella, Haemophilus, Neisseria, Granulicatella, and Fusobacterium, undergo shifts in diversity during esophageal inflammation or in patients with BE, showing decreased Streptococcus levels and increased proportions of G**-** anaerobic/microaerophilic bacteria (24). BE significantly escalates the risk of developing EAC, potentially thirtyfold, with microbiota alterations contributing to this progression (30). Surface-associated lipopolysaccharides (LPS) from Gram-negative bacteria trigger the NF-κB inflammatory signaling pathway, releasing a plethora of inflammatory mediators that may foster cancer development. Multi-omics analyses underscore the pivotal role of local esophageal microbiota in the progression from GERD to esophageal metaplasia (31). Metaplastic samples are rich in Helicobacter species, mirroring findings in BE patients (24, 27). Pathway analysis has revealed upregulation in bile acid secretion, cAMP signaling, lysosomal processing, and other cancer-related pathways. Co-culturing isolated Helicobacter strains with allogeneic human macrophages and subsequent cytokine release assays highlight sustained inflammation in infected macrophages for up to 18 hours compared to controls. Compared to those with BE, the esophageal microbiota of EAC patients exhibits reduced diversity, diminished Veillonella and Granulicatella levels, with Lactobacilli becoming more dominant, thriving in the low pH environment induced by chronic inflammation in the gastric cardia region (32). Research indicates that lactic acid bacteria (LAB) such as Lactobacillus, Bifidobacterium, and Streptococcus are prevalent in EAC tissues, with Lactobacillus identified as a critical bacterium in EAC (33, 34). Recent findings by Snider et al. suggest that increased levels of Akkermansia muciniphila and Enterobacteriaceae, alongside reduced Veillonella, may facilitate EAC development in patients with high-grade dysplasia or EAC (35). This suggests a complex interplay between microbial interactions and risk factors for BE and EAC, highlighting the mutual reinforcement and association between them.

Notably, Helicobacter pylori has been recognized as a carcinogen linked to the progression of various gastric disorders, such as gastritis, gastric ulcers, atrophy, and adenocarcinoma. Although it primarily colonizes the gastric mucosa, its presence also impacts the microbial composition of the lower esophagus. Tian et al. have shown that while Helicobacter pylori does not replicate within the esophagus, it can significantly affect the diversity of the esophageal microbiota (28). Additionally, several studies have observed a lower incidence of EAC in individuals infected with Helicobacter pylori than in those uninfected (36, 37), suggesting that Helicobacter pylori infection may exert a protective effect against the development of EAC. The underlying mechanisms for this potential protective effect are not fully elucidated, although several plausible hypotheses have emerged from current research. One hypothesis posits that Helicobacter pylori infection may lead to atrophic gastritis and decreased gastric acid secretion (38), which in turn could reduce the acidity of gastric reflux. Reduced reflux acidity may lessen the risk of GERD, a recognized precursor to BE and subsequent EAC. Further, some studies have observed that the eradication of Helicobacter pylori might increase serum ghrelin levels, which could contribute to obesity and affect gastric emptying, potentially triggering the onset of BE and EAC. Helicobacter pylori may also confer protection by inducing apoptosis in EAC cells via activation of the Fas-caspase signaling pathway (37). Despite these possible protective effects, it is critical to recognize Helicobacter pylori as a Class I human carcinogen, closely linked to various gastric pathologies including gastritis, gastric ulcers, gastric atrophy, and gastric adenocarcinoma. The bacterium’s virulence factors, such as CagA, VacA, and adhesins, are known to drive chronic inflammation and carcinogenesis, activating inflammatory responses through pathways like NF-κB and promoting the secretion of **i**nflammatory mediators such as IL-1β, IL-2, and TNF-α within the gastric epithelium (39). Additionally, Helicobacter pylori can directly damage DNA, disrupt key transcription factors like Cdx2, and alter acid secretion, further contributing to epithelial injury (40). Another proposed mechanism of its carcinogenic action includes elevating gastrin levels, a hormone implicated in the growth of gastrointestinal tumors and potentially in the progression from BE to EAC (41). While some meta-analyses and studies support an inverse correlation between Helicobacter pylori infection and EAC, these findings are contradicted by other studies, and many have confounding variables that complicate the understanding of EAC pathogenesis. This underscores the need for more rigorously designed prospective cohort studies to further investigate the relationship between Helicobacter pylori and EAC, including its potential therapeutic implications. Addressing the feasibility of manipulating the gastrointestinal microbiota to promote Helicobacter pylori colonization in EAC patients without incurring associated risks remains a complex and challenging endeavor. While it is recognized that Helicobacter pylori is a major risk factor for gastric cancer, particularly strains harboring the cag pathogenicity island and the CagA protein, the fact that most Helicobacter pylori-infected individuals do not develop gastric cancer suggests the involvement of other mitigating factors. Enhancing Helicobacter pylori colonization could lead to increased chronic inflammation, heightened risks of ulcers and cancer, antibiotic resistance, and adverse effects on the gastrointestinal microbiota. Therefore, although it is theoretically conceivable to augment Helicobacter pylori colonization by modulating the microbiota, such a strategy entails significant risks and necessitates extensive research to evaluate its feasibility and safety.

### 2.3 Intestinal microbiota profile in EC

Alterations in gut microbiota can substantially impact EC development. Münch et al. used a mouse model of BE to show that a high-fat diet induced esophageal dysplasia by modifying the tissue microenvironment and gut microbiota, promoting inflammation and stem cell growth (42). The changes in microbial composition triggered by the eating pattern were linked to increased production of inflammatory mediators and heightened immune activity, creating a tumor-promoting environment. This highlights the pivotal role of gut microbiota in mediating diet-related inflammatory responses. Deng et al. analyzed the gut microbiome profiles of EC patients and healthy individuals by performing 16S amplicon sequencing on stool samples. Their findings revealed a higher abundance of Firmicutes and Actinobacteria, alongside a reduction in Bacteroidetes, in the gut microbiota of EC patients. They also noted a reduction in SCFAs-producing microbes and a rise in LPS-producing microbes among the EC group (43). SCFAs, such as acetate, propionate, and butyrate, play crucial roles in regulating inflammation, cellular processes, and apoptosis, and are essential for bacterial energy metabolism and gut health. These molecules also function as signaling agents with significant anticarcinogenic effects, whereas dysbiosis is linked to the development of gastrointestinal cancers, including EC (44). Further studies have confirmed differences in fecal microbiota between cancer patients and healthy controls. Analysis of stool samples from individuals with esophageal, gastric, and colorectal cancers revealed distinct microbiome profiles when compared to healthy subjects (45). Cancer patients exhibited an increased abundance of Bacteroides fragilis, Escherichia coli, Akkermansia muciniphila, Clostridium hathewayi, and Alistipes finegoldii, while healthy individuals had higher levels of Roseburia faecis, Clostridium clostridioforme, Faecalibacterium prausnitzii, Bifidobacterium adolescentis, Blautia producta, and Butyricicoccus pullicaecorum. A recent meta-analysis found that EC patients exhibited an increased Chao1 index but a reduced Shannon index. At the phylum level, there was a significant decrease in Firmicutes and a notable increase in Bacteroidetes and Proteobacteria. At the genus/family level, the abundance of Bacteroidaceae, Prevotellaceae, and Streptococcaceae was markedly lower, whereas Veillonellaceae showed a significant increase (46). These findings emphasize the link between gut microbiota composition and gastrointestinal cancers, suggesting promising diagnostic and therapeutic opportunities.

## 3 Mechanisms of microbiota-mediated development in esophageal carcinogenesis

**Figure 1:**
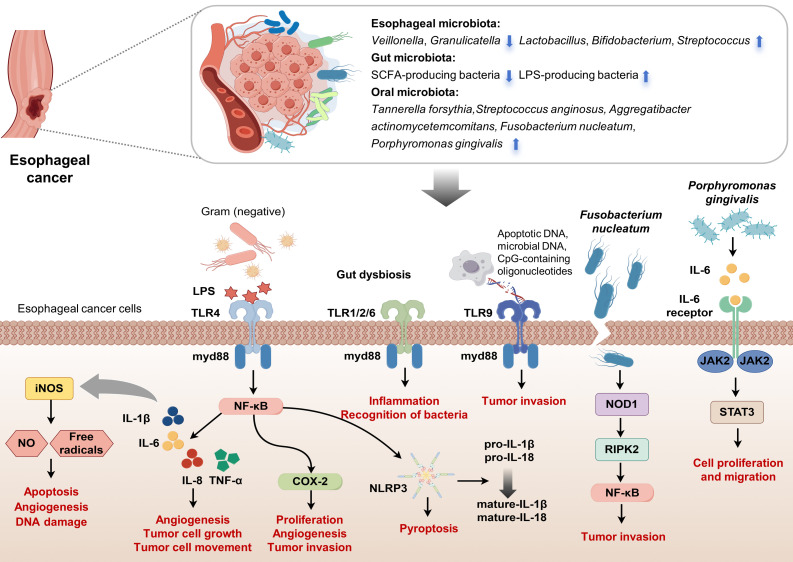
Interactions between microbiota and esophageal carcinogenesis. This figure visually summarizes the complex interactions between various microbiota and their impact on EC pathogenesis, focusing on ESCC. It highlights esophageal microbiota dysbiosis, with pathogenic bacteria activating carcinogenic pathways. The diagram also depicts beneficial SCFAs-producing bacteria and harmful LPS-producing bacteria within the gut microbiota. Oral microbiota, including Tannerella forsythia, Streptococcus anginosus, Aggregatibacter actinomycetemcomitans, Fusobacterium mucleatum and Porphyromonas gingivalis, influence EC development. Central to the figure is the signaling cascade initiated by bacterial LPS interacting with TLRs on esophageal cancer cells. LPS from Gram-negative bacteria activates TLR4, while other microbial components stimulate TLR1/2/6 and TLR9, signaling through the adaptor protein MyD88. This activation leads to downstream inflammatory responses via the NF-κB pathway, inducing cytokines (IL-1β, IL-6, IL-8, TNF-α) that promote tumor cell proliferation, angiogenesis, invasion, and the expression of COX-2 and NLRP3, which are involved in inflammation and pyroptosis. The figure also illustrates how Fusobacterium nucleatum and Porphyromonas gingivalis specifically contribute to EC progression. Porphyromonas gingivalis interacts with the IL-6 receptor, activating JAK2/STAT3 signaling, which drives cell proliferation and migration. Fusobacterium nucleatum promotes inflammation and tumor invasion via NF-κB and NOD-like receptor signaling pathways. The interplay between these microbial factors and the host immune response underscores the potential of the microbiota as targets for cancer therapy and diagnostics. (Drawn by FigDraw).

The esophageal microbiota, an essential component of the tumor microenvironment in EC, is closely linked to dysplastic changes in the esophageal squamous epithelium and may significantly contribute to EC development (**Figure 1**). Studies involving ESCC patients have reported decreased levels of Bacteroidetes, Fusobacteria, and Spirochaetes, as well as reduced microbial diversity in tumor tissues (47). Similarly, patients with high-grade dysplasia and EAC show microbiome shifts during the progression from BE to EAC, characterized by an increase in Enterobacteriaceae and mucin-adherent Akkermansia muciniphila, along with a reduction in Veillonella species. Understanding the microbiota’s role in esophageal carcinogenesis could provide important insights for developing therapeutic strategies. Chronic inflammation from gastroesophageal reflux often initiates EAC, promoting intestinal metaplasia and inflammatory cytokine production, with immune cells playing a central role in this progression. Microbial changes in BE may further accelerate EAC development through persistent inflammation (48). Under normal circumstances, innate immune cells detect antigens such as LPS, peptidoglycan, or flagellin via TLRs, activating the MyD88-dependent pathway (49), which results in cytokine and interferon production, thereby promoting adaptive immunity. However, an imbalance in microbial composition can impair immunity and activate inflammatory pathways that may lead to cancer. In reflux esophagitis, increased expression of TLR4 on esophageal epithelial cells, caused by heightened exposure to pathogen-associated molecular patterns, results in activation of the NF-κB pathway, which raises pro-inflammatory mediators levels (50–52). This pathway activation is associated with the progression from reflux esophagitis to BE and adenocarcinoma, with an elevation in leukocyte interleukins (IL-1β and IL-6 etc.) (53). Activation of TLR4 also stimulates cyclooxygenase-2, implicated in the development of EAC (54), and its inhibition has been shown to prevent reflux-induced adenocarcinoma. The role of cytokine IL-8 in carcinogenesis includes modulating angiogenesis, cell growth, mobility, and immune responses (55), with evidence suggesting that elevated TLR4 expression correlates with poor prognosis in EAC. Moreover, increased expression of TLR2, which can interact with other TLRs to recognize a broader array of ligands, has been observed in EAC tumors. The activation of the TLR1/2/6 network in BE and cancer enhances inflammation and microbial recognition by precancerous cells. The role of TLR9, associated with advanced cancer stages and poor outcomes (56), underscores the complex interactions between microbial DNA and cancer cell invasiveness, highlighting the need for further investigation into how esophageal microbiota composition directly influences TLR expression and cancer progression. Additionally, LPS and inflammatory cytokines trigger the expression of iNOS, leading to the production of NO and free radicals that promote apoptosis, angiogenesis, and DNA damage during tumorigenesis (57). NO also upregulates matrix metalloproteinases and tissue inhibitor of metalloproteinases (TIMPs), with TIMPs facilitating the progression of BE from dysplasia to invasive carcinoma (58). Elevated iNOS expression is observed in BE and EAC compared to normal esophageal tissues. Immunohistochemical analysis indicates significantly higher iNOS levels in BE patients than in gastric controls. Additionally, microbial imbalances lead to the secretion of cytokines IL-1β and IL-18 and activate caspase-1 via the NLRP3 inflammasome. Studies show that LPS stimulation activates the NLRP3 inflammasome downstream of TLR4 by upregulating NLRP3, pro-IL-1β, and pro-IL-18 expressions. Moreover, LPS enhances mitochondrial ROS production, triggering activation of the NLRP3 inflammasome, which results in pyroptosis and the maturation of pro-IL-1β and pro-IL-18 into their active forms (59).

Fusobacterium nucleatum in EC tissues is associated with shorter survival and promotes tumor invasion by activating chemokines such as CCL20. Transmission electron microscopy studies reveal that Fusobacterium nucleatum invades and proliferates within ESCC cells, affecting gene and protein expression levels. Analysis shows an enrichment in the NF-κB and NOD-like receptor signaling pathways in ESCC cells treated with Fusobacterium nucleatum. These cells display enhanced growth capabilities and increased NF-κB activation, along with elevated levels of NOD1 and phosphorylated RIPK2, which accelerates tumor growth in xenograft models (60). Liang et al. found that Fusobacterium nucleatum also promotes the enrichment of immunosuppressive MDSCs through activation of the NLRP3 inflammasome, contributing to chemoresistance in ESCC by influencing autophagy (61, 62). In female NSG mice inoculated with ESCC tissue, Fusobacterium nucleatum infection resulted in increased MDSCs and enhanced resistance to cisplatin (61). Higher levels of Fusobacterium nucleatum were found in the saliva of ESCC patients compared to controls, correlating with more advanced disease stages and poorer chemotherapy response (63, 64). In parallel, Porphyromonas gingivalis has been implicated in promoting ESCC progression. Studies using cell lines show that Porphyromonas gingivalis activates the NF-κB signaling pathway, enhancing cell proliferation and motility (13). In animal models, Porphyromonas gingivalis infection correlates with advanced disease stages and poorer outcomes, mediated through the IL-6-STAT3 pathway (65). This bacterium also confers chemotherapy resistance, further complicating treatment efforts (66). Elevated salivary levels of Porphyromonas gingivalis in ESCC patients, compared with healthy counterparts, indicate a significant positive association with disease severity and adverse prognosis (20, 63, 66). Lastly, the role of lactate in EAC tumorigenesis is increasingly recognized. Lactate acts as a critical signaling molecule in tumor metabolism, influencing angiogenesis, immune evasion, cell migration, and metastasis (67). Studies of microbial communities in GERD and BE esophagi show elevated lactate production, predominantly by lactate-producing bacteria such as Staphylococci, Lactobacilli, Bifidobacteria, and Streptococci (33). The increased presence of these bacteria in gastric adenocarcinoma necessitates further investigation into the specific impacts of lactate on cancer progression and its potential therapeutic implications.

## 4 Utilizing the microbiome as a biomarker for EC Diagnosis

Compared to non-cancerous esophageal tissues, the alpha diversity of high-grade intraepithelial neoplasia and adenocarcinoma is significantly diminished. Observations also reveal a decrease in Firmicutes and an increase in Proteobacteria as BE progresses to EAC. In studies of oral microbiota in BE patients, marked taxonomic differences are evident, including increased levels of Streptococcus, Veillonella, and Enterobacteriaceae, alongside reduced levels of Neisseria, Corynebacterium and Lautropia. Notably, the combination of Streptococcus, Lautropia, and a genus in the order Bacteroidales has demonstrated high diagnostic accuracy for identifying BE patients, achieving a sensitivity of 96.9% and specificity of 88.2% (68). Research in China indicates significant reductions in Neisseria in patients with ESCC compared to controls, while Veillonella and Prevotella are notably enriched, suggesting their potential as novel biomarkers for EC detection (69). Liu et al. analyzed the esophageal microbiota of ESCC patients across various stages to identify microbial biomarkers for prognosis. Their findings revealed that lymph node-positive patients had increased levels of Bacteroidetes, and Spirochaetes, and Firmicutes, while Proteobacteria was less abundant in those without lymph node involvement. At the genus level, Treponema and Prevotella were more prevalent in patients with lymph node metastasis, whereas Streptococcus was enriched in advanced tumor stages. A higher combined presence of Streptococcus and Prevotella correlated with worse survival outcomes, suggesting their role as prognostic indicators for ESCC (70). Fusobacterium nucleatum has been associated with unfavorable prognoses in colorectal cancer. Yamamura et al. analyzed Fusobacterium nucleatum DNA using qPCR in EC specimens and found that its presence correlated with reduced survival, suggesting its potential role as a prognostic biomarker. Furthermore, high levels of Fusobacterium nucleatum are linked with an increased risk of recurrence and resistance to neoadjuvant chemotherapy, underscoring its significance as a predictor of poor treatment outcomes (19, 64).

Liu et al. utilized 16S amplicon sequencing to evaluate the oral microbiome in pre-diagnostic oral specimens, highlighting the significant role of oral microbiome composition in EC, thereby emphasizing its potential as an early detection biomarker (71). Li et al. examined paired biopsy and swab specimens from patients across different pathological conditions, revealing that microbial diversity in swab specimens closely mirrored that of the esophageal mucosa. The co-occurrence of Streptococcus and Neisseria appeared to predict the progression of ESCC effectively (72). Further, a study involving participants undergoing endoscopic examination found diminished microbial diversity in saliva and brush samples as the disease progressed. This analysis identified shared microbial biomarkers across various stages of dysplasia and EC, highlighting the presence of Granulicatella, Rothia, Streptococcus, Gemella, Leptotrichia, and Schaalia in low-grade dysplasia, with Lactobacillus prevailing in high-grade dysplasia. Notably, EC patients displayed distinct biomarkers including Bosea, Solobacterium, Gemella, and Peptostreptococcus, and the top 3 microbial signatures in the saliva and cell brush specimens provided high diagnostic accuracy, as reflected in the area under the curve values of 87.16% and 89.13% (73). Lastly, Deng et al. analyzed the gut microbiota from EC and found that Lachnospira, Streptococcus, Bacteroides, and Bifidobacterium had high diagnostic accuracy for EC, with areas under the curve exceeding 0.85 (43). Notably, Lachnospira emerged as a particularly promising potential biomarker for distinguishing EC patients from healthy individuals. Although these findings mark an initial identification of potential bacterial biomarkers for EC, there are several crucial challenges that need addressing. These challenges include variability in study designs, differences in detection methods, inconsistent control groups, and the risk of sample contamination. Consequently, it is imperative to conduct a comprehensive multi-center study involving a large sample size and a broad cohort, employing standardized methods to further validate and identify reliable bacterial biomarkers for EC diagnosis.

**Figure 2:**
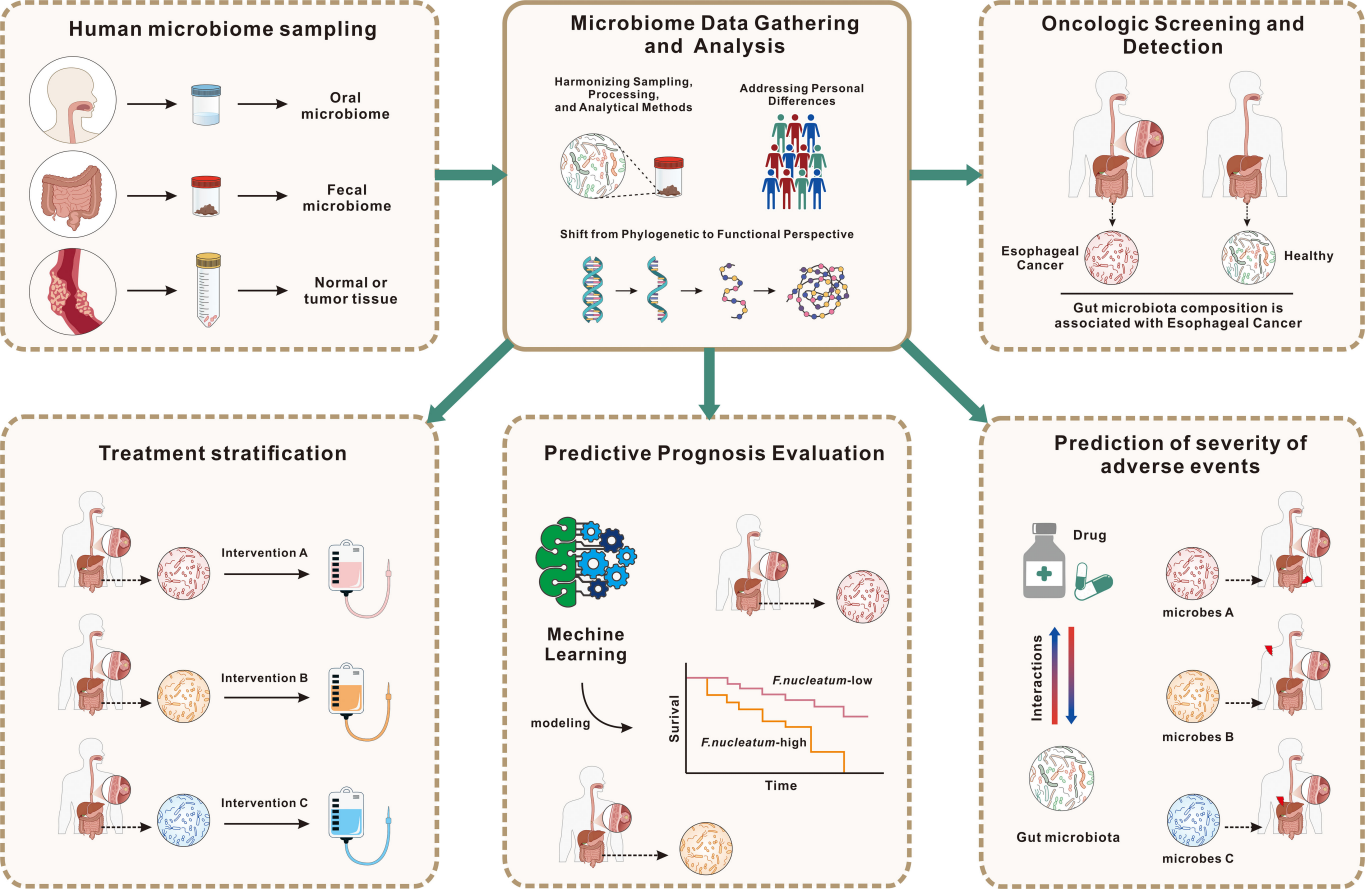
Application of gut microbiota data for EC diagnosis, prognosis, and patient stratification. This figure illustrates the utilization of gut microbiota data to enhance the diagnosis, prognosis, and treatment of EC. The process begins with microbiome sampling from oral, fecal, and tumor tissues, followed by standardized data collection and analysis, considering individual variability. Gut microbiota profiles are used for oncologic screening, aiding in distinguishing EC patients from healthy individuals, and for prognosis prediction, employing machine learning models to estimate survival outcomes based on microbial composition, such as F. nucleatum levels. Additionally, microbiota characteristics can predict adverse treatment effects, informing risk management. Microbial data also support treatment stratification, guiding personalized interventions to optimize therapeutic efficacy. This highlights the potential of microbiome profiling in advancing EC patient care through precision medicine.

Circulating microbiome DNA (cmDNA) is gaining attention in cancer biomarker research due to the increasing acknowledgment of the microbiome’s involvement in cancer. Chen et al. have introduced cmDNA as an innovative liquid biopsy technique for cancer diagnosis (74), involving the detection of microbial genomic DNA in serum, which reflects the systemic circulation of microbes. cmDNA comprises DNA from various sources, including bacteria, viruses, fungi, and parasites, with bacterial DNA being the most abundant due to the low overall microbial biomass in serum. Microbial DNA can either be released endogenously from deceased microbes or actively produced by commensals. The persistence of cmDNA in the bloodstream is facilitated by an impaired immune clearance mechanism. In cancer patients, weakened immune responses allow cmDNA to remain in circulation. Additionally, cmDNA can be encapsulated within host-derived outer membrane vesicles (OMVs), enabling it to evade immune surveillance. This encapsulation suggests that cmDNA within OMVs could provide a comprehensive snapshot of the human microbiome, representing a promising new approach for cancer biomarker identification. The potential applications of cmDNA in cancer diagnostics are extensive, ranging from population-wide early screening to the precise differentiation of cancer subtypes and distinguishing between benign and malignant conditions (75). For instance, serum microbiome DNA analysis in gastric cancer patients has shown a reduction in alpha diversity, with certain species emerging as potential diagnostic indicators (76). Serum microbial genomic profiles have also been instrumental in classifying subtypes of myeloid malignancy, enhancing the clinical relevance of microbial analyses (77). Metagenomic analysis of microbe-derived OMVs in serum has led to the formulation of a diagnostic model capable of differentiating ovarian cancer from benign ovarian tumors (78). And Chen et al. developed a cmDNA-based diagnostic model with high sensitivity and accuracy, capable of detecting early-stage lung cancer and predicting recurrence post-surgery (79). Although histopathological analysis remains the definitive method for cancer staging, molecular biomarkers, including those derived from the microbiome, are increasingly critical, enabling earlier diagnosis and more precise classification of cancer subtypes, as well as staging and prognosis prediction. Future research should focus on assessing the synergistic effects of gut microbiota and other biomarkers. The primary aim is to establish a methodology that leverages gut microbiota for the early detection of EC, monitoring of metastasis, and optimization of treatments (**Figure 2**).

## 5 Microbiota’s impact on therapeutic efficacy and resistance in EC

### 5.1 Microbiota’s effect on chemotherapy effectiveness

Given that gut microbiota can influence tumor growth and treatment response, assessing gut microbiota before chemotherapy may help predict the efficacy of chemotherapeutic drugs, thus providing a stronger basis for selecting treatment plans. A study on patients with locally advanced rectal cancer found that evaluating gut microbiota could predict the effectiveness of neoadjuvant chemoradiotherapy (nCRT). Results showed a significant reduction in pathogenic bacteria and an increase in beneficial bacteria, such as Lactobacillus and Streptococcus, which correlated with treatment response (80). Bingula et al. reported notable changes in the intestinal flora of elderly non-small cell lung cancer patients after chemotherapy and found that the immune response of specific memory Th1 cells to Enterococcus and Enterobacter pasteurella could predict progression-free survival in advanced lung cancer patients, highlighting the role of gut microbiota in chemotherapy efficacy. Microbiota-derived metabolites, such as indole-3-acetic acid, have been shown to enhance chemotherapy efficacy in pancreatic cancer by promoting oxidative stress in cancer cells, thereby compromising their metabolic fitness and proliferation (81). The gut microbiota can also modify immune responses during chemotherapy. Cyclophosphamide, a commonly used chemotherapeutic agent, has been shown to alter gut microbiota composition, resulting in the translocation of specific bacterial species that activate antitumor immune responses (82). This indicates that the microbiota not only affects drug metabolism but also plays a key role in shaping the immune environment during cancer treatment. Additionally, specific microbes such as Streptococcus and Prevotella in the esophageal microbiome of ESCC patients serve as independent predictors of poor outcomes (70). Roseburia faecis, affected by chemotherapy, shows promise as a biomarker for treatment efficacy in EC (45). Fusobacterium nucleatum, commonly found in the oral and gastrointestinal tracts, is associated with cancer progression and chemotherapy resistance in ESCC (83). High intratumoral levels of Fusobacterium nucleatum correlate with increased recurrence, shorter relapse-free survival, and poorer response to neoadjuvant chemotherapy in EC (64). The link between the gut microbial community and tumor metastasis in EC further underscores the potential of microbial biomarkers in predicting disease spread (84). Maintaining a balanced gut microbiota is therefore essential for optimizing chemotherapy in cancer patients. These findings emphasize the gastrointestinal microbiome’s critical role in influencing chemotherapy outcomes, predicting prognosis, and guiding treatment strategies in EC.

The nematode Caenorhabditis elegans has been instrumental as a model organism for understanding the complex interactions between hosts and their microbiota, particularly in cancer treatment contexts. Research has shown that specific bacteria can significantly influence the host’s response to chemotherapeutic agents. For example, different bacterial species can enhance or reduce the efficacy of drugs like 5-fluorouracil and 5-fluoro-2-deoxyuridine, commonly used in EC treatments, through mechanisms involving bacterial metabolism (85, 86). Notably, in colorectal cancer (CRC), Fusobacterium nucleatum has been known to bolster chemotherapy resistance by either promoting autophagy within cancer cells or by enhancing the expression of BIRC3, which inhibits apoptosis (87). Given the similarities in chemotherapy protocols between CRC and EC, these mechanisms could also influence EC. Additionally, retrospective studies in CRC have indicated that antibiotics might boost the effectiveness of oxaliplatin, though such effects are not seen with irinotecan (88). In a mouse model of lung cancer, Lactobacillus was shown to enhance cisplatin efficacy. Compared to mice treated only with cisplatin, those receiving Lactobacillus alongside cisplatin exhibited reduced tumor volume and extended survival. Additionally, levels of the immunosuppressive cytokine IL-10 were lower in the combination treatment group. These results suggest that microbiota modulation might also improve chemotherapy outcomes in EC. Moreover, Geller et al. demonstrated that Gammaproteobacteria in CRC can metabolize gemcitabine into an inactive form, leading to drug resistance (89). Irinotecan, a common chemotherapeutic agent, is metabolized by gut bacteria, which can lead to both therapeutic effects and adverse reactions such as diarrhea (90). Probiotics have been explored as a means to mitigate these side effects by restoring a healthy gut microbiome, thereby enhancing the overall treatment experience for patients (90, 91). The interaction between microbiota and chemotherapy effectiveness highlights the importance of further research into microbiome-targeted therapies. A deeper understanding of this relationship could improve treatment protocols, minimize side effects, and enhance patient quality of life during EC therapy.

### 5.2 Microbiota’s impact on immunotherapy effectiveness

EC is typically diagnosed at an advanced or metastatic stage, resulting in poor survival outcomes. The immune landscape in EC shows notable changes compared to a healthy esophagus. In BE, a precursor to EAC, T cells develop a Th2 phenotype, and PD-L2 expression is elevated (92). In EAC, there is a shift to a mixed Th1/Th2 phenotype, with increased Tregs and myeloid-derived suppressor cells (93). CD8+ tumor-infiltrating lymphocytes are associated with better survival, while higher HLA-DR expression in epithelial cells correlates with poorer survival. In ESCC, CD8+ T cell infiltration is linked to improved outcomes, whereas alternatively activated macrophages are linked to worse prognosis (94). The advent of immunotherapy, particularly through immune checkpoint inhibitors (ICIs), has dramatically transformed cancer treatment. Immune checkpoints are regulatory molecules on immune cells that control the extent of immune activation, thus preventing an overactive immune response. Key immune checkpoints include PD-1, LAG3, TIM3, CTLA-4, and TIGIT. PD-1/PD-L1 inhibitors and CTLA-4 inhibitors are the most frequently used ICIs in clinical settings, targeting crucial pathways in T cell activation and exhaustion, thereby serving as pivotal regulators of anti-tumor T cell activity. The current evidence positions ICIs as fundamental in combating EC, marking a new era in cancer treatment paradigms. The influence of the microbiome on the efficacy of ICIs in EC remains under-researched. The significance of the gut microbiome in enhancing the efficacy of immunotherapies, particularly PD-1/PD-L1 antibody treatments, is well established for early-stage cancers including melanoma, non-small cell lung cancer, and gastrointestinal malignancies. Yet, its role in enhancing PD-1 antibody therapy for EC, especially during neoadjuvant treatment phases, has not been extensively explored (95–97). Notably, Understanding the mechanisms through which the gut microbiota affects immune responses is crucial for improving ICIs efficacy. Studies indicate that the gut microbiome can downregulate immune checkpoint pathways, such as PD-L2 and its binding partner RGMb, thereby promoting a more robust anti-tumor response (98). Bifidobacterium has been linked to improved antitumor immunity and enhanced response to PD-L1 blockade therapy (99). Additionally, modulation of the gut microbiota through dietary changes or fecal microbiota transplantation has shown promise in overcoming resistance to ICIs and improving patient outcomes (100, 101). Under inflammatory conditions, Campylobacter concisus has been found to increase PD-L1 expression in epithelial cells (102). Furthermore, a phase 1 clinical trial has been initiated to explore the effects of fecal microbiota transplantation (FMT) on patients with gastrointestinal tumors treated with anti-PD-1 therapies (NCT04130763). Interim results indicate that FMT is well-tolerated and that the microbiome profiles of responders are more similar to those of the donors compared to nonresponders, mirroring findings from another recent FMT trial in melanoma (103). And recent studies have scrutinized the gut microbiota of ESCC patients undergoing surgery post-neoadjuvant PD-1/PD-L1 antibody therapy and chemotherapy. These studies primarily focused on assessing pathological complete response (pCR) and major pathological response (MPR), with fecal samples collected at key treatment milestones. Observations revealed a marked reduction in microbiome diversity following the combination therapy, and notably, microbiome profiles prior to treatment differed significantly between patients who achieved pCR and those who did not (74). ICIs can induce gastrointestinal immune-related adverse events (irAEs) such as diarrhea and colitis, which may require discontinuation of treatment. Microbiome composition variations have been associated with the occurrence and severity of irAEs, suggesting that gut microbiota could potentially predict both irAEs and overall treatment response (104, 105). Interestingly, antibiotic use during anti-PD-1/PD-L1 or combined anti-CTLA-4 and PD-1/PD-L1 therapies in advanced esophagogastric cancer patients has not shown the typical adverse effects observed in lung, renal, or melanoma cancers (106, 107). This indicates that the microbiome’s role in ICIs therapy for EC may differ from other cancers, warranting further investigation. Continued exploration of the microbiome-immunotherapy relationship may pave the way for individualized oncological strategies based on patients’ unique microbiome profiles (104).

## 6 Advanced microbiota management: pioneering personalized treatment in EC

**Figure 3:**
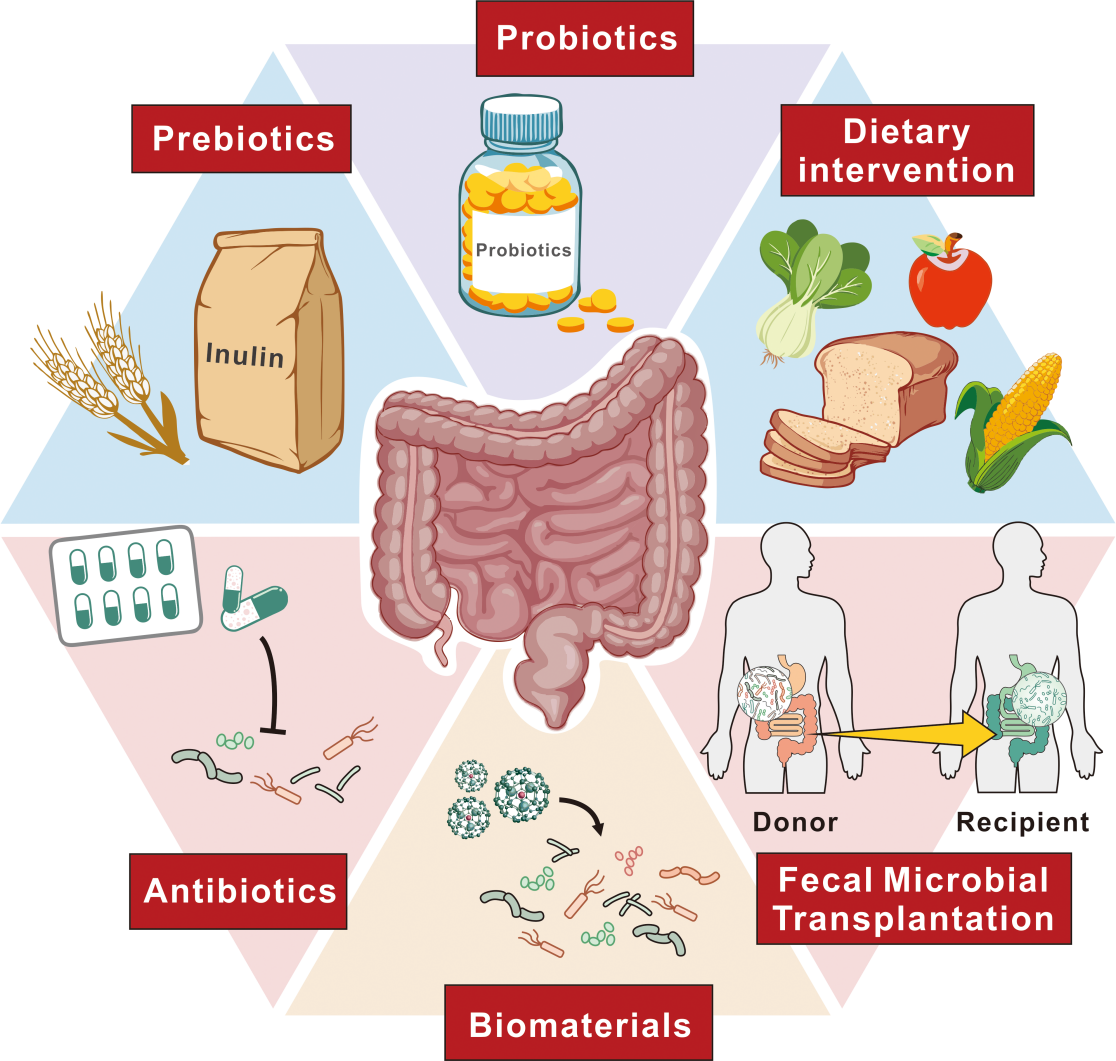
Approaches to alter gut microbiota for EC therapy. This figure outlines various strategies to modify the gut microbiota as a therapeutic approach for EC. These approaches include probiotics, which introduce beneficial microorganisms, and prebiotics, such as inulin, which promote the growth of beneficial gut bacteria. Dietary interventions aim to support a balanced microbiota through specific food choices. Antibiotics are used to selectively modulate bacterial populations, while FMT involves transferring healthy donor microbiota to recipients to restore a balanced microbial environment. Additionally, biomaterials are employed to facilitate targeted modulation of microbial communities. Together, these strategies represent potential avenues to improve treatment outcomes for EC patients through microbiome modulation.

Precise microbiota modulation is emerging as a key strategy in enhancing treatment protocols for EC. This approach is based on the understanding of how specific microbial taxa influence carcinogenesis and therapeutic outcomes, incorporating individual microbiota data into precision medicine to better predict which patients might benefit from particular treatments. Recognizing microbiota dysbiosis as a pathological condition is essential, underscoring the need for increased awareness among physicians and patients about its implications.The complexity of cancer pathogenesis and the diversity of clinical outcomes in EC are shaped by the interplay between cancer-associated and host factors, reflecting the heterogeneity of the disease. To effectively manipulate the gut microbiota for therapeutic benefit, several strategies are utilized, including dietary modifications, probiotics, FMT, and antibiotics. These methods are designed to modify the gut microbiota composition to enhance the efficacy of treatments. Furthermore, nanomedicine offers an interdisciplinary approach that targets specific pathogenic bacteria, improving the precision of microbial interventions in cancer therapy. This comprehensive strategy, by adjusting the microbiota, has the potential to revolutionize EC therapy, leading to more personalized and effective treatment outcomes (**Figure 3**).

Although popular, dietary interventions often face issues with inconsistent adherence without close supervision. Currently, no standardized dietary management protocol exists for cancer patients. Yet, emerging practices like periodic fasting mimicking diets (FMD) show potential as supplements to various cancer therapies, including immunotherapy. A clinical trial with 101 cancer patients found that a five-day FMD combined with standard therapy is safe and feasible, reshaping anticancer immunity by reducing immunosuppressive cells and enhancing immune responses linked to better outcomes (108). One umbrella review suggests that adherence to the Mediterranean diet may reduce breast cancer risk, particularly in postmenopausal women (109). And intermittent fasting and the Mediterranean diet positively influence gut microbiota and may aid cancer prevention and treatment, whereas evidence for the ketogenic diet is less conclusive (108). Research into dietary components shows that certain foods can impact the makeup and population of specific gut bacteria. This relationship between diet and gut microbiota is crucial, as the gut microbiota plays a significant role in human health and disease. For instance, dietary fibers such as inulin, resistant starch, and citrus pectin have been shown to selectively modulate the gut microbiota composition, leading to beneficial metabolic changes (110). These dietary fibers can promote the growth of beneficial bacteria while reducing the abundance of potentially harmful ones, thereby enhancing gut health. Moreover, the interaction between food components, particularly polyphenols, and gut microbiota has garnered attention in recent years. Research indicates that the food matrix and processing methods can influence how these phytochemicals interact with gut bacteria, ultimately affecting their bioavailability and health benefits (111). This two-way interaction highlights the importance of considering not just the nutrients themselves but also how they are presented in food. Further, dietary protein type and amount have been shown to influence gut microbiota composition in preclinical and clinical studies. Excessive protein consumption can lead to dysbiosis, characterized by an imbalance in microbial communities, which is associated with various health issues. Conversely, moderate protein intake, particularly from sources rich in beneficial amino acids and peptides, may support a healthy gut microbiome and promote the production of beneficial metabolites (112). Diets high in animal protein have been associated with an increase in certain pathogenic bacteria, while plant-based proteins may promote a more diverse and beneficial microbiota (113). Moreover, the interplay between dietary proteins and microbes can also affect the synthesis of microbial metabolites, which have been implicated in cancer development. Understanding how specific protein sources influence microbial composition and function could provide insights into dietary strategies for cancer prevention.

Research on probiotics in cancer treatment has largely focused on their potential to enhance immune function, thereby aiding in combating cancer. For example, during the perioperative period in CRC patients, Lactobacillus gallinarum has been shown to adhere to the colonic mucosa, reducing fecal pathogen levels and modulating local immune responses (114). Akkermansia muciniphila has demonstrated efficacy in restoring the response to PD-1 inhibitors in mice (106). Similarly, Bifidobacterium may boost antitumor immunity and improve the effectiveness of anti-PD-L1 therapies (99). Additionally, specific probiotics like Lactobacillus acidophilus LAC-361 and Bifidobacterium longum BB-536 have been linked to reduced radiation-induced diarrhea, highlighting their role in alleviating side effects of radiation therapy (115). These instances highlight the considerable promise of probiotics in cancer treatment. Nonetheless, thorough evaluation of their long-term safety is mandatory before clinical application, particularly in terms of diverse diets and treatments. Moreover, FMT is being explored as a supplemental therapy to restore gut microbiota in cancer patients. While it may not benefit those with tumor-specific resistance, identifying microbiota elements that influence ICI efficacy is crucial. Research should refine FMT protocols, focusing on delivery methods, frequency, duration, and antibiotic preconditioning. Techniques like transendoscopic enteral tubing may facilitate repeated transplants and sampling. Improved donor and recipient selection is needed for better outcomes, and understanding gut and tumor mechanisms is essential. Advances in metagenomics enhance tracking microbiota and metabolites, with monitoring serving as a biomarker for treatment efficacy. Despite progress, FMT’s low acceptance requires public awareness and standardized procedures to increase adoption.

Biotechnological advances are refining the precision of microbiota manipulation in esophageal tissue and solid tumors, offering new therapeutic avenues for EC. Innovations such as narrow-spectrum antibiotics, bacteriophages, and antibody-directed therapies are critical in targeting and neutralizing harmful microbes or bacteria-encoded genes, thus facilitating the selective eradication of deleterious microbes without significantly altering the overall microbiota composition. Synthetic biology could further revolutionize this field by engineering bacteria to act as “smart probiotics,” designed specifically for targeted drug delivery. Techniques to control the movement and release of therapeutic agents from engineered bacteria, through the manipulation of physical properties like temperature or magnetism, can significantly reduce unintended collateral damage outside tumor tissues (116). Nanomaterials are increasingly utilized as vehicles to transport therapeutic agents directly to targeted sites. This method enhances drug stability, extends their presence in the bloodstream, and mitigates adverse effects by reducing unintended drug deposition in non-target tissues (117, 118). Taking advantage of these properties, nanomedicines are crafted to selectively neutralize pathogenic microbes. The targeting capabilities of bacteriophages are particularly notable in this regard, as they can specifically eliminate harmful microorganisms. Building on this approach, Zhang et al. created a targeted nanomedicine to combat Fusobacterium nucleatum, which plays a significant role in the initiation and progression of CRC (119). They used a bacteriophage specifically engineered to infect Fusobacterium nucleatum, modified with azide groups, and encapsulated anti-CRC drugs within dextran nanoparticles. These drugs were subsequently covalently linked to the azide-modified phages, ensuring that the phage-mediated nanoparticles precisely target Fusobacterium nucleatum. This bacteriophage-based nanomedicine allows for selective modulation of gut microbiota, significantly enhancing the efficacy of chemotherapy in CRC treatment. Additionally, OMVs derived from engineered bacteria provide a versatile platform for carrying tumor-specific antigens, facilitating targeted immune responses. Li et al. emploied OMVs engineered with L7Ae and listeriolysin O (OMV-LL) as an mRNA delivery platform for antitumor vaccination. OMV-LL efficiently delivers mRNA antigens to DCs, significantly inhibiting tumor growth and inducing long-term immune memory (120). This approach offers a promising alternative to lipid nanoparticles for personalized mRNA vaccines.

## 7 Concluding remarks and microbial insights in EC

Microbial imbalance is increasingly recognized as a critical factor in esophageal carcinogenesis, particularly in ESCC. Advances in microbial sequencing have expanded our understanding, but establishing causal links between specific microbes and EC remains challenging. Dysbiosis involving bacteria such as Porphyromonas gingivalis and Fusobacterium nucleatum contributes to the progression of EC and resistance to chemotherapy through activation of pathways like TLR4-NF-κB and IL-6-STAT3. Higher-resolution sequencing beyond the genus level is necessary to accurately identify bacterial contributions, while future research should also consider the roles of viruses, fungi, and microbial metabolites, as they could serve as diagnostic biomarkers and therapeutic targets. Dietary and environmental factors significantly influence the esophageal microbiota, affecting cancer onset and progression. However, variability in microbial data across studies highlights the need for standardized sampling protocols, and innovations such as non-endoscopic sampling capsules may provide minimally invasive solutions. The potential of oral microbiota as a biomarker for ESCC is promising, though prospective validation is needed. Future research should also integrate multi-omics approaches, including metabolomics, proteomics, and metatranscriptomics, to fully elucidate the functional characteristics of the microbiota. Examining the influence of diet and medications is important for understanding their roles in EC development. Regulating the intestinal microbiota presents an opportunity to improve cancer outcomes, with interventions like FMT, probiotics, or antibiotics showing promise in modulating the tumor microenvironment and enhancing anti-cancer immunity. Nonetheless, comprehensive clinical trials and animal model studies are needed to evaluate these strategies and unravel the mechanisms underlying microbiota-driven carcinogenesis, ultimately paving the way for more effective treatments.
